# Novel green UPLC method with life cycle assessment for determination of favipiravir and molnupiravir drugs and environmental water samples

**DOI:** 10.1038/s41598-026-41131-z

**Published:** 2026-03-31

**Authors:** Khadiga Mohamed Kelani, Menna S. Elsherbiny, Sherif M. Eid, Maha F. Abdel Ghany, Mahmoud M. Abbas

**Affiliations:** 1 Analytical Chemistry Department, Faculty of Pharmacy, Cairo University, El-Kasr El-Aini Street, Cairo, 11562 Egypt; 2 Analytical Chemistry Department, Faculty of Pharmacy, Modern University for Technology and Information, Cairo, Egypt; 3 Analytical Chemistry Department, Faculty of Pharmacy, October 6 University, 6 October City, Giza Egypt; 4 Pharmaceutical Analytical Chemistry Department, Faculty of Pharmacy, Ain Shams University, Abbassia, Cairo, 11566 Egypt; 5 Department of Medicinal Chemistry, Faculty of Pharmacy, Galala University, New Galala City, 43713 Suez Egypt

**Keywords:** Favipiravir, Molnupiravir, UPLC, Solid-Phase Extraction, Green Analytical Chemistry, Life Cycle Assessment, Chemistry, Environmental sciences

## Abstract

A novel green UPLC–SPE analytical method was developed for the simultaneous determination of Favipiravir (FVP) and Molnupiravir (MPV) in pharmaceutical and environmental water samples. The method was designed to address the analytical gap surrounding concurrent antiviral monitoring given their co-administration while minimizing solvent consumption, waste generation, and environmental impact. Water samples preparation employed a solid-phase extraction strategy optimized through systematic evaluation of sorbent type, conditioning, washing, and elution steps. Chromatographic separation was achieved using a mobile phase consisting of 0.01 M potassium dihydrogen phosphate buffer (pH 3.5) and 50% methanol (85:15, v/v), delivered at a flow rate of 0.2 mL/min with a 10 µL injection volume. This method produced sharp, well-resolved peaks with a total run time of less than 5 min. The method exhibited excellent linearity (r² ≥ 0.999), accuracy (98.56–101.19%), precision (RSD ≤ 1.8%), and recoveries of (98.97- 100.74%) across tap water, Nile River water, and pharmaceutical wastewater. Greenness was assessed using the Multi-Color Assessment (MA) platform, yielding a final whiteness score of 85.7%, which indicates a well-balanced integration of environmental, practical, analytical, and innovative attributes and supports the method’s suitability for routine application. The Analytical Eco-Scale score (77) and AGREE pictogram score (0.84) further confirm the strong environmental compatibility of the proposed method. Life cycle assessment (LCA) revealed substantial reductions in energy consumption and waste generation compared with conventional HPLC methods. Overall, the proposed UPLC–SPE approach, integrated with LCA and comprehensive greenness evaluation, represents a pioneering and environmentally informed strategy for antiviral analysis and provides a sustainable platform for future pharmaceutical residue monitoring.

## Introduction

Human innovation has enabled major societal advances, with pharmaceuticals—especially antiviral drugs—among the most influential. These compounds treat a broad spectrum of viral infections, from mild illnesses to severe diseases such as hepatitis C, H1N1 influenza, and COVID-19^1,2^. The COVID-19 pandemic triggered a sharp rise in global antiviral use, with increases of up to 37% in some regions^3^. Consequently, higher levels of antiviral residues now enter the environment through human excretion and pharmaceutical manufacturing effluents. Their presence in aquatic systems raises concerns about ecological toxicity, human re-exposure via insufficiently treated wastewater, and the emergence of antiviral-resistant viruses—already observed in influenza strains^4,5^.

**Fig. 1:**
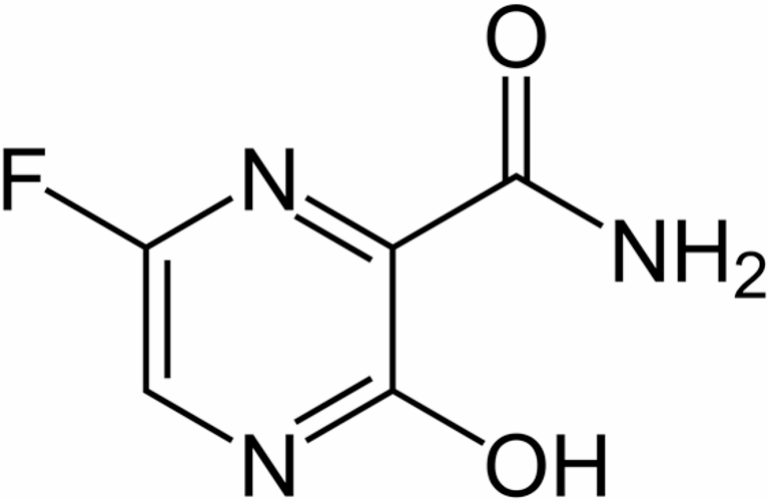
Structural formula of favipiravir (FVP).

**Fig. 2:**
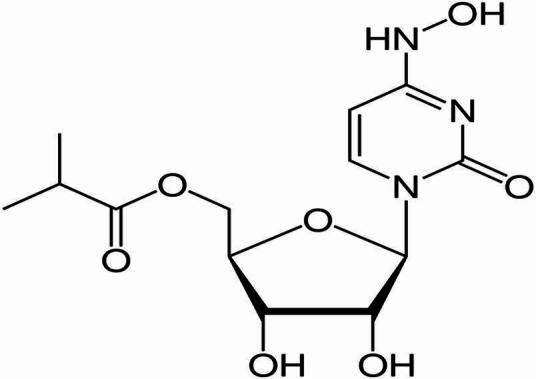
Structural formula of Molnupiravir (MPV).

Favipiravir (FVP) is a broad-spectrum antiviral that inhibits RNA-dependent RNA polymerase, blocking viral replication. Originally developed for influenza, it was later assessed for Ebola treatment and has demonstrated activity against coronaviruses (Fig. 1)^6^. Molnupiravir is a prodrug that, once metabolized, incorporates into viral RNA and induces lethal mutagenesis, producing non-viable viral particles (Fig. 2)^7,8^. It became the first orally administered antiviral authorized for COVID-19 treatment, receiving emergency use authorization in the United Kingdom in 2021^9^.

The widespread post-pandemic use of FVP and MPV, coupled with their incomplete metabolism and continuous discharge into wastewater, has raised concerns regarding their occurrence, persistence, and ecological effects. Their environmental accumulation may contribute to ecological toxicity and antiviral resistance. Despite their growing relevance, analytical research remains limited—especially regarding simultaneous quantification of both compounds in environmental matrices and the sustainability of the analytical methods employed. Most existing methods rely on acetonitrile-rich mobile phases, long chromatographic runs, or conventional sample-preparation techniques, with little consideration for environmental sustainability. Few studies apply green analytical chemistry principles, and fewer still incorporate life cycle assessment (LCA) to evaluate impacts related to solvent use, energy consumption, and waste generation. These gaps highlight the need for robust, sensitive, and environmentally responsible analytical methods capable of simultaneously detecting both antivirals in complex matrices.

To address these gaps, the present work introduces a green UPLC–SPE workflow for the simultaneous determination of FVP and MPV in pharmaceutical and environmental water samples, using ethanol–water as a greener solvent system. The method’s environmental performance was evaluated using Analytical Eco-Scale, AGREE, and a cradle-to-grave life cycle assessment (LCA) that included solvent production, instrument operation, and waste disposal.

Since the pandemic, research on commonly used antiviral agents has expanded, and FVP–MPV combinations—often co-administered to enhance therapeutic outcomes^10^—have been analyzed using spectroscopic^11,12^, chromatographic^13–15^, and electrochemical methods^16^. However, no validated protocols have yet been reported for detecting these antivirals in environmental samples, despite mounting evidence of their occurrence in aquatic environments^17,18^.

The authors previously developed a fluorometric method for the simultaneous analysis of FVP and MPV in pharmaceutical products and environmental samples^19^. Building on this foundation, we now introduce a green ultra-performance liquid chromatography (UPLC) method for their simultaneous determination, coupled with a comprehensive LCA. LCA evaluates environmental impacts across a process’s full life cycle—from raw material extraction to end-of-life disposal. While LCA was conceptualized in the 1960s, it was standardized by the International Organization for Standardization (ISO) in the late 1990s^20,21^. Growing global emphasis on environmental sustainability has expanded its adoption, and many regulatory bodies now encourage or require LCA-based evaluations^22^. Despite this, its application in analytical chemistry remains limited. Incorporating LCA into method development can guide more sustainable laboratory practices and inform decision-making.

UPLC was selected as the model technique for LCA implementation because chromatographic methods are widely used in pharmaceutical analysis and are associated with substantial solvent consumption, energy demand, and waste generation—characteristics that make them ideal targets for environmental optimization. LCA provides a structured framework for identifying environmental hotspots and developing strategies to reduce impacts throughout the analytical workflow.

In summary, this study aims to develop and validate a green UPLC–SPE method for the simultaneous determination of favipiravir and molnupiravir in pharmaceutical formulations and environmental water samples, while comprehensively evaluating its environmental performance using the Multi-Color Assessment (MA) tool for whiteness assessment, the Analytical Eco-Scale, AGREE, and detailed life cycle assessment (LCA) tools.

## Experimental

### Goal and scope of the life cycle assessment (LCA)

The primary objective of the life cycle assessment (LCA) was to evaluate the environmental impact of a newly developed green analytical method utilizing ultra-performance liquid chromatography coupled with solid-phase extraction (UPLC-SPE) for the simultaneous determination of Favipiravir and Molnupiravir in pharmaceutical and environmental samples. The LCA aims to guide greener analytical method development by identifying environmental hotspots and supporting decisions aligned with green chemistry principles.

The functional unit for this study was defined as the analysis of one sample (pharmaceutical or environmental) using the developed UPLC-SPE method. This unit provides a consistent basis for comparing alternative analytical workflows or solvent systems.

#### System components and life cycle stages

The life cycle of the UPLC-SPE method was assessed using a cradle-to-grave model and comprises the following stages:

- Transport: Collection and transportation of environmental samples from the source to the laboratory.
- Use Phase: Sample preparation, SPE procedures, UPLC operation, energy consumption, and solvent use during chromatographic separation.
- End-of-Life: Disposal of consumables including used SPE cartridges, solvents, vials, syringe filters, and chromatographic columns.

#### System boundaries

The system boundary encompasses all processes directly related to the analytical method. Included within the boundary are:

- Energy consumption associated with SPE and UPLC analysis, including instrument operation and supporting systems (e.g., Heating, Ventilation, and Air Conditioning, known as HVAC, if relevant^20–22^.
- Waste generation and disposal resulting from solvent usage, cartridges, filters, and columns.
- Single-use and reusable consumables (e.g., pipette tips, vials, filters).
- Transportation of environmental samples.

The following components were excluded from the analysis due to data unavailability or limited relevance: energy associated with laboratory infrastructure, instrument manufacturing (due to long instrument lifespan), and personnel-related energy contributions.

#### Impact assessment methodology

Environmental impacts were quantified using the ReCiPe Midpoint (H) method^23^, which encompasses 17 midpoint indicators contributing to three endpoint damage categories:

- Human Health (e.g., human toxicity, particulate matter formation, ozone depletion),
- Ecosystems/Biodiversity (e.g., marine, freshwater, and terrestrial ecotoxicity),
- Resource Availability (e.g., fossil resource scarcity, water use).

This method provides a comprehensive framework for characterizing the environmental burden associated with each life cycle stage of the analytical process.

#### Green analytical chemistry optimizations

Several strategies were implemented to align the analytical method with the principles of green analytical chemistry:

- Solvent Selection: Preference for water-rich mobile phases with reduced reliance on hazardous solvents such as acetonitrile.
- Solvent Volume Reduction: Optimization of SPE and chromatographic steps to minimize solvent usage.
- Shortened Analysis Time: Reduced run times to lower energy demand and solvent consumption.
- Efficient Use of Materials: Employment of durable UPLC columns and exploration of reusable or recyclable SPE cartridges.
- Miniaturization and Automation: Consideration of micro-scale SPE and automated systems to reduce variability, resource consumption, and labor intensity.

#### Green SPE method development

Recognizing that solid-phase extraction is both critical to analytical performance and a major contributor to environmental impact, efforts were made to improve its sustainability:

- Use of reduced solvent volumes for conditioning, washing, and elution.
- Exploration of alternative green solvents such as ethanol and water.
- Implementation of low-energy alternatives for drying and concentration (e.g., ambient air-drying instead of nitrogen blow-down).
- Evaluation of cartridge reusability and recyclable formats.
- Consideration of on-line SPE integration to reduce sample handling and associated waste.

### Chemicals and reagents

Pure standard powders of Favipiravir (FVP, 99.65%) and Molnupiravir (MPV, > 98%) were obtained from Sigma-Aldrich (Germany). The purity of both standards was further confirmed using a previously reported validated spectroscopic method^12^.

Commercial pharmaceutical formulations were procured from local pharmacies in Cairo, Egypt. Avipiravir tablets contain 200 mg of favipiravir per tablet, while Molnupiravir capsules contain 200 mg of molnupiravir per capsule.

A phosphate buffer solution (pH 3.5) was prepared using potassium dihydrogen phosphate and orthophosphoric acid. The mobile phase consisted of methanol (50%), the prepared buffer, and bi-distilled deionized water. All reagents and solvents were of analytical grade and were obtained from Fisher Scientific UK Ltd.

All chemicals were used in the preparation of the mobile phase, with the exception of ethyl acetate, which was utilized exclusively during the solid-phase extraction (SPE) process.

### Instruments and software

Chromatographic separation was performed using an Agilent 1200 Series UPLC–UV system (Agilent Technologies, USA) equipped with an autosampler and a variable wavelength UV–VIS detector. Separation was achieved on a Kinetex C18 column (100 Å, 2.6 μm, 4.6 mm × 100 mm).

Data acquisition and processing were carried out using Agilent ChemStation software, version A.10.01.

For sample preparation, a P10 single-channel micropipette (set to 10 µL) was used. pH measurements were verified using a Jenway 3505 pH meter (Jenway, UK).

Solid-phase extraction (SPE) was conducted using Bakerbond SPE C18 cartridges (Fisher Scientific, UK) in conjunction with a vacuum manifold system.

The MA Tool platform was developed using modern web technologies, including HTML5, CSS3, and JavaScript (ES6+), ensuring cross-platform compatibility and optimal performance across all major web browsers (Chrome, Firefox, Safari, and Edge). The application is hosted on the Netlify cloud platform and is freely accessible at https://effervescent-naiad-a47bbd.netlify.app, requiring no installation or registration.

### Stock and working solutions

Accurately weighed quantities of 0.05 g of pure Favipiravir (FVP) and Molnupiravir (MPV) were each transferred into separate 50 mL volumetric flasks. The powders were dissolved using distilled water to prepare stock solutions with a final concentration of 1000 µg/mL for each drug.

To obtain the working solutions (100 µg/mL), 5 mL of each stock solution was accurately pipetted into separate 50 mL volumetric flasks, and the volume was completed to the mark with distilled water. The flasks were inverted several times to ensure complete homogenization.

## Method development and optimization

### Chromatographic conditions

The method development for the simultaneous determination of Favipiravir (FVP) and Molnupiravir (MPV) was guided by the physicochemical properties of both analytes, prior analytical findings, and the principles of green analytical chemistry. FVP and MPV are polar nucleoside analogues with relatively low log P values and partial ionization in mildly acidic conditions, making reversed-phase chromatography appropriate when using an aqueous buffer capable of stabilizing their retention.

#### Chromatographic method development

A series of experiments was conducted to evaluate the effects of pH, buffer type, and organic modifier on chromatographic separation. Buffer pH values of 3.0, 3.5, and 4.0 were investigated, as the ionization behavior of both antiviral agents markedly influences retention and peak symmetry. Among the tested conditions, a phosphate buffer at pH 3.5 provided the best overall performance, affording enhanced resolution and more symmetrical peaks. This finding is in agreement with previous reports showing that acidic phosphate buffers improve chromatographic stability and peak shape for nucleoside analogues^24^.

Mobile-phase optimization was guided by principles of green analytical chemistry. Hazardous solvents such as acetonitrile and butanol were intentionally excluded. Although alternative, greener alcohols were examined, they failed to deliver satisfactory chromatographic performance for the simultaneous separation of FVP and MPV. Methanol, which is less toxic and more biodegradable than acetonitrile, demonstrated superior retention behavior and peak shape. Accordingly, the optimized mobile phase comprised 0.01 M phosphate buffer and methanol in an 85:15 (v/v) ratio, with the pH adjusted to 3.5 using orthophosphoric acid.

Separation was carried out on a Kinetex C18 column (100 Å, 2.6 μm, 4.6 × 100 mm), which provided sufficient hydrophobic interaction to retain both analytes while maintaining short analysis times. The optimized chromatographic conditions employed isocratic elution, a flow rate of 0.2 mL/min, an injection volume of 10 µL, and a column temperature of 30 °C. Detection at 230 nm using a UV–VIS detector provided adequate sensitivity and reproducibility for both antiviral compounds.

These conditions achieved efficient separation while also minimizing solvent consumption due to the low flow rate and absence of more hazardous organic solvents, aligning the method with environmentally conscious analytical practices.

### Construction of calibration curves

Two sets of 10 mL volumetric flasks were used to prepare serial dilutions of Favipiravir (FVP) and Molnupiravir (MPV) for calibration curve construction. Specified volumes of the respective 100 µg/mL working solutions were accurately pipetted into the flasks and diluted to volume using the mobile phase.

The resulting calibration concentrations ranged from 0.3 to 25 µg/mL for both FVP and MPV. From each prepared solution, 10 µL was injected into the UPLC system under the optimized chromatographic conditions. The corresponding chromatograms were recorded, and the average peak area for each concentration was calculated from triplicate injections.

Calibration curves were constructed by plotting the average peak area against the corresponding drug concentration for each analyte. The resulting data were fitted using linear regression analysis, and the regression equations were subsequently determined for both FVP and MPV.

### Analysis of laboratory-prepared mixtures

To assess the accuracy and selectivity of the proposed method in complex matrices, laboratory-prepared mixtures of Favipiravir (FVP) and Molnupiravir (MPV) were analyzed.

Accurately measured volumes of the FVP working solution were transferred into a series of 10 mL volumetric flasks, followed by the addition of appropriate volumes of the MPV working solution to achieve the following final concentration ratios (FVP: MPV, µg/mL): 2:20, 6:14, 8:12, and 12:8.

Each flask was filled to the mark with the mobile phase and thoroughly mixed to ensure complete homogenization of both analytes.

The prepared mixtures were then analyzed according to the UPLC procedure described in Sect.  2.5.2, and the average peak areas were used to determine the concentrations of FVP and MPV using the previously constructed calibration curves.

### Application to pharmaceutical formulations

Ten units each of Avipiravir tablets and Molnupiravir capsules were used. A quantity equivalent to the weight of one tablet and one capsule was accurately weighed and employed to prepare stock solutions with concentrations of 2000 µg/mL for Favipiravir (FVP) and Molnupiravir (MPV), respectively. The powdered dosage forms were dispersed in distilled water, sonicated to ensure complete dissolution, then filtered to remove any insoluble excipients.

These stock solutions were then appropriately diluted with distilled water to obtain working solutions at a concentration of 1 µg/mL for each drug. All volumetric preparations—both stock and working solutions—were made up to the mark with distilled water.

Subsequently, five different concentrations within the established linearity range were prepared by appropriate dilution in 10-mL volumetric flasks, using the mobile phase as the diluent. Each solution was brought to volume and thoroughly mixed by inversion to ensure homogeneity prior to analysis.

### Application to environmental water samples (solid-phase extraction)

Environmental water samples were collected from various sources, including tap water, Nile River water, and industrial wastewater. Tap water samples were gathered from multiple locations across Cairo and Giza. Representative Nile River water samples were obtained from Giza, Egypt, using clean, sterilized containers. Sampling was performed by immersing inverted containers just below the water surface until filled, followed by immediate sealing and labeling with detailed collection information^25^.

Industrial wastewater samples were collected from a pharmaceutical manufacturing facility located in Cairo. All samples were transported in temperature-controlled containers to the analytical laboratory. To ensure sample integrity, processing and analysis were conducted on the same day as collection.

Initially, the three environmental water samples were analyzed without spiking to assess the presence of Favipiravir (FVP) and Molnupiravir (MPV) in the natural matrix and it was found that no FVP or MPV were detected above their LOD values. Subsequently, samples were spiked with pure FVP and MPV at three different concentrations to validate the analytical method. The water samples were subjected to solid-phase extraction (SPE) prior to UPLC analysis to enhance sample purity.

Conditioning:

The SPE procedure began with conditioning the Bakerbond SPE C18 cartridge (Thermo Fisher Scientific) by passing 6 mL of 50% methanol through the stationary phase (the sorbent) using a vacuum manifold. The vacuum was released as the methanol approached the top of the sorbent bed to prevent drying. The cartridge was then equilibrated with 3 mL of distilled water to maintain proper wetting of the stationary phase.

Loading:

For sample loading, 1 mL of each spiked water sample was applied to the preconditioned SPE cartridge, and the filtrate was collected in a test tube.

Washing:

After loading, the cartridge was washed with distilled water to remove residual matrix components while preserving analyte retention.

Elution:

The analytes (FVP and MPV) retained on the sorbent were subsequently eluted with ethyl acetate, and the eluate was dried using a rotary evaporator. The resulting residue was reconstituted in 10 mL of the mobile phase^26^, followed by filtration through a 0.45 μm membrane filter (Cosmonice Filter S, Nacalaitesque) prior to chromatographic analysis under the conditions described in Sect.  2.5.2.

According to solvent selection guidelines, methanol and ethyl acetate are categorized as acceptable alternatives when greener solvents are insufficient to dissolve or extract the target compounds effectively^27^. Furthermore, several studies indicate that combining these solvents with water—rather than using them in their pure forms—can significantly reduce their environmental impact^28,29^.

In this study, although water and ethanol were initially tested, methanol and ethyl acetate were ultimately selected due to their superior extraction performance and favorable rankings among green solvents.

Method validation:

All validation procedures were carried out according to the International Council for Harmonization (ICH) guidelines^30^.

## Results and discussion

This study presents a novel, environmentally friendly UPLC method for the simultaneous determination of Favipiravir (FVP) and Molnupiravir (MPV).

### Chromatographic optimization of the method

Method development for simultaneous determination of FVP and MPV was guided by the physicochemical properties of both analytes, prior analytical findings, and the principles of green analytical chemistry. FVP and MPV are polar nucleoside analogues with relatively low log P values and partial ionization in mildly acidic conditions, making reversed-phase chromatography appropriate when using an aqueous buffer capable of stabilizing their retention.

The choice of mobile-phase and extraction media was driven by their favorable environmental profiles, compatibility with the physicochemical properties of the target analytes, and alignment with the principles of green analytical chemistry. Although water and ethanol were initially tested, methanol and ethyl acetate were ultimately selected because they provided superior extraction performance and are considered among the greener options within their solvent classes. According to solvent selection guidelines, both methanol and ethyl acetate are classified as acceptable alternatives when greener solvents cannot adequately dissolve or extract the target compounds effectively^27^. Additionally, several studies have shown that using these solvents in combination with water - rather than using them pure form - substantially reduces their overall environmental impact^28,29^.

A range of chromatographic conditions were evaluated to achieve the best possible sensitivity and resolution while preserving the method’s overall greenness. Phosphate buffer, methanol, and micellar mobile phases containing sodium dodecyl sulfate were tested at different flow rates and at various mobile-phase ratios (50:50, 70:30, 80:20, and 85:15), along with wavelength scans between 230 and 250 nm. Ultimately, a green mobile phase was selected, consisting of 0.01 M potassium dihydrogen phosphate buffer and 50% methanol in an 85:15 ratios, with a flow rate of 0.2 mL/min. The buffer pH was adjusted to 3.5 using orthophosphoric acid to ensure optimal separation.

Phosphate buffer was chosen as the major component (85%) of the mobile phase to minimize the use of less environmentally friendly solvents like methanol, thereby enhancing the overall greenness of the method. Additionally, phosphate buffer can be safely disposed of after dilution with water, as it is initially prepared using distilled water. The injection volume was set at 10 µL.

**Fig. 3:**
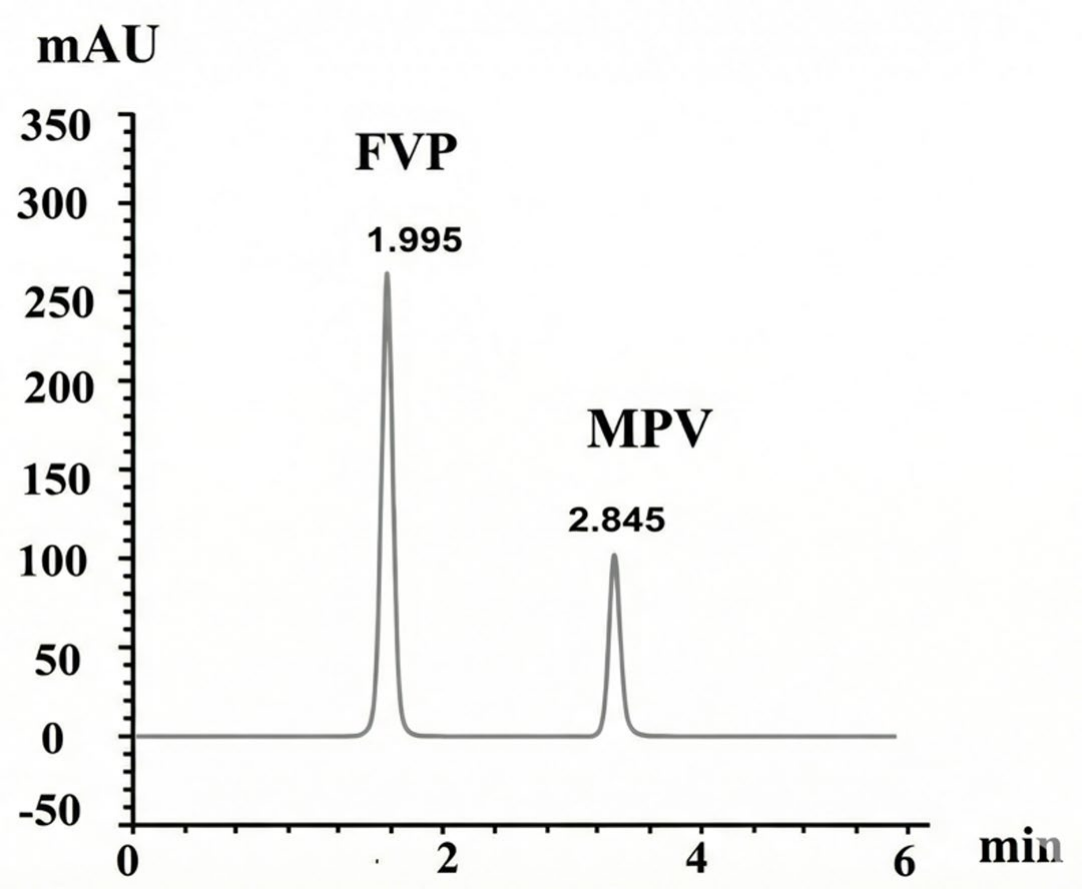
UPLC chromatogram of Pure FVP (10 µg/mL) at RT = 1.995 min and Pure MPV (10 µg/mL) at RT = 2.845 min.

Under these optimized conditions, the retention times were 1.995 min for pure FVP and 2.833 min for pure MPV, with a resolution of 7.14 between the two peaks, as illustrated in Fig. 3. The optimal detection wavelength was determined to be 230 nm using a UV-Vis detector. 

**Fig. 4:**
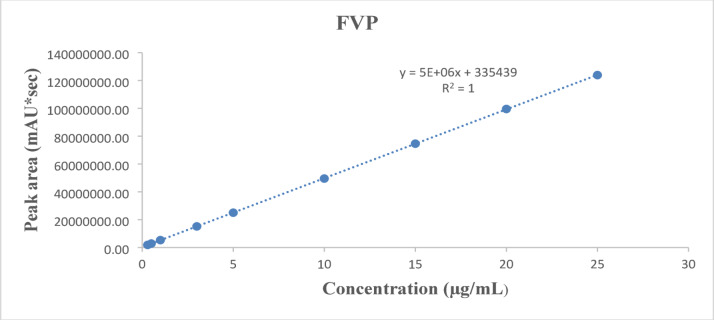
Linearity of FVP relating peak area to concentration (0.3–25 µg/mL) using the UPLC-SPE method.

**Fig. 5:**
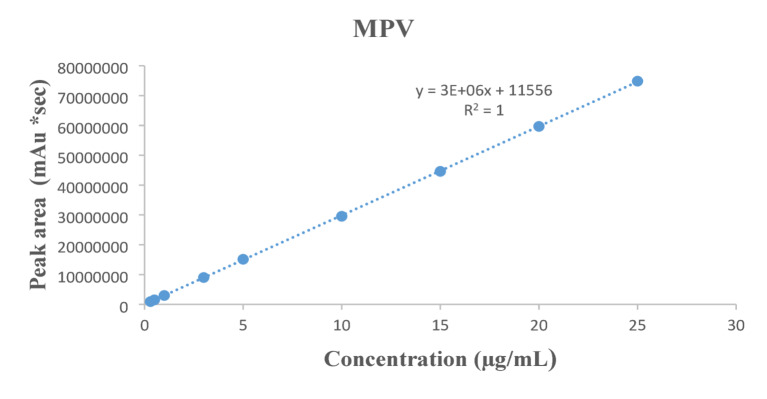
Linearity of MPV relating the peak area to concentration (0.3–25 µg/mL) using the UPLC-SPE method.

The calibration curves showed excellent linearity, with the regression equations:

- For FVP: A = 5 × 10^6^ C + 335,439 (Fig. 4).
- For MPV: A = 3 × 10^6^ C + 11,566 (Fig. 5).

where A is the peak area and C is the concentration in µg/mL.

### Method validation

The method was validated following the updated ICH Q2(R2) (2023) guideline^30^. Linearity was confirmed over clinically and environmentally relevant concentration ranges with r² ≥ 0.999 for both analytes. Accuracy studies showed recoveries between 98.3 and 101.5%, and precision (intra-day and inter-day) yielded RSD ≤ 1.8%. Robustness testing confirmed minimal variation when flow rate, pH, and detection wavelength were slightly altered.

#### Linearity

The linearity of FVP and MPV was determined over the concentration range of 0.3 to 25 µg/for both drugs, following the procedures described in Sect. 2.5.2. Calibration curves were constructed, and the regression parameters were computed. Also, the corresponding recoveries for both drugs were calculated, the results are summarized in Table 1.

**Table 1:** Construction of calibration curves of pure samples of FVP and MPV by the proposed UPLC-SPE method.

Sample FVP (µg/mL)	Found* (µg/mL)	% Recovery	Sample MPV (µg/mL)	Found* (µg/mL)	% Recovery
0.3	0.31	102.60	0.3	0.31	103.16
0.5	0.50	100.66	0.5	0.51	101.44
1	1.00	99.66	1	0.99	99.46
3	2.97	98.91	3	3.00	100.09
5	4.94	98.72	5	5.04	100.81
10	9.84	98.42	10	9.84	98.44
15	14.85	99.03	15	14.86	99.09
20	19.84	99.20	20	19.89	99.44
25	24.71	98.84	25	24.94	99.77
Mean ± SD		98.56 ± 1.32	Mean ± SD		100.19 ± 1.43

*Average of 3 determinations.

#### Accuracy

The accuracy of the method was assessed by calculating the average percentage recovery (%R) for FVP and MPV. Each concentration level was analyzed in triplicate using UPLC, and the average value of the three measurements was used for recovery calculation. The results are presented in Table 2.

**Table 2:** Regression parameters and validation steps of FVP and MPV using the proposed UPLC-SPE method.

Parameter	FVP	MPV
Wavelength (nm)	230	230
Linearity’s and validation steps		
Slope	5E + 06	3E + 06
Intercept	335,439	11,556
Correlation coefficient (r^2^)	1	1
Range (µg/mL)	0.3–25	0.3–25
Accuracy *(M* ± SD*)*	98.56 ± 1.32	100.19 ± 1.43
LOD (µg/mL)	0.05	0.09
LOQ (µg/mL)	0.15	0.28
Precision (%RSD):		
Repeatability	0.65	1.03
Intermediate Precision	0.80	1.19
Specificity: *(mean ± RSD)*	99.12 ± 0.80	99.12 ± 0.67
Robustness (% RSD)		
pH (± 0.1)	0.87	1.51
Flow rate (± 0.02 mL/min)	0.97	1.58

#### Precision

Repeatability and intermediate precision were evaluated by analyzing three selected concentrations of each drug three times at different short intervals within the same day. While the intermediate precision was assessed by analyzing the same three concentrations on three separate days within the same week. The results are presented in Table 2.

#### Robustness

Small deliberate variations in method parameters (specifically flow rate and pH) were introduced to assess method robustness. Relative standard deviations were then calculated, and no differences were observed in the results, confirming the robustness and reliability of the method under slight changes in analytical conditions (Table 2).

#### System suitability parameters

System suitability parameters were evaluated according to the United States Pharmacopeia (USP) guidelines^31^; the results are summarized in Table 3.

**Table 3:** System suitability parameters for the determination of FVP and MPV using the UPLC-SPE method.

Parameters	Obtained value		Reference value^31*^
	FVP	MPV	
Resolution (R)	7.14		*R* > 0.8
Capacity factor (K)	2.71	2.45	1–10 acceptable
Selectivity factor (α)	1.11		> 1
Number of theoretical plates (N)	6545	5875	Increases with increasing the efficiency of separation
Tailing factor (T)	0.88	1.08	T ≤ 2 T = 1 for a typical symmetric peak
Height equivalent to one theoretical plate (H) (cm)	0.08	0.11	The smaller the value, the higher the column efficacy

*U.S. Pharmacopeial Convention. (n.d.). Chromatography (General Chapter < 621> ), 2022. 10.31003/USPNF_M99380_06_01^31^.

#### Specificity

Lab-prepared mixtures of FVP and MPV were formulated and analyzed according to the procedures mentioned in Sect.  **2.5.2** to further validate the method and confirm its specificity. The results obtained are shown in Table 4.

**Table 4:** Determination of favipiravir (FVP) and molnupiravir (MPV) in laboratory prepared mixtures using the proposed method.

Added FVP (µg/mL)	Found* (µg/mL)	% Recovery	Added MPV (µg/mL)	Found* (µg/mL)	%Recovery
2	1.98	98.94	20	19.67	98.33
6	6.02	100.29	14	13.84	98.85
8	7.89	98.68	12	11.98	99.81
12	11.83	98.59	8	7.96	99.50
Mean %R ± SD		99.12 ± 0.80	Mean %R ± SD		99.12 ± 0.67

*Mean of three experiments.

#### Application of the standard addition technique

The validated procedure was applied to commercial pharmaceutical formulations, namely Avipiravir tablets and Molnupiravir capsules. To further validate the accuracy of the method, the standard addition technique was employed; the results obtained are presented in Table 5.

**Table 5:** Application of standard addition techniques for determination of FVP in Avipiravir tablets and MPV in Molnupiravir capsules using the proposed UPLC method.

Drug	Pharmaceutical taken (µg/mL)	Pharmaceutical found* (µg/mL)	Pure added (µg/mL)	Pure found* (µg/mL)	Pure recovery (%*R*)
FVP	6	5.96	1	1.05	101.25
			3	2.92	98.62
	3	2.89	5	4.87	98.43
			9	8.83	98.59
	Mean %R ± SD	96.33 ± 0.98	99.22 ± 1.36		
MPV	6	5.94	1	0.99	99.75
			3	2.93	98.81
	3	2.92	5	5.02	100.21
			9	8.94	99.52
	Mean %R ± SD	98.17 ± 0.79	99.57 ± 0.59		

*Average of 3 determination.

#### **Statistical comparison with previously reported methods**

The method was statistically compared to previously reported methods and no significant difference was observed based on t-test and F-test results, confirming the reliability of the developed approach (Table 6).

**Table 6:** Statistical comparison between the results obtained by the proposed UPLC-SPE method and reported methods.

Parameters	Proposed method		Reported method*^12^	
	FVP	MPV	FVP	MPV
Number of measurements	9	9	5	5
Mean % recovery	99.56	100.19	99.99	99.99
% RSD	1.32	1.43	0.82	1.23
Variance	1.72	2.04	1.34	0.44
Student’s t-test**	0.61 (2.18)	0.29 (2.18)		
F-value**	1.28 (4.82)	4.63 (6.04)		

*Reported method for MPV determination is an HPLC method with UV detection at 230 nm^12^.

**The values in parenthesis are tabulated values of “*t”* and “*F at P = 0.05.*

### Application

#### Application to pharmaceutical dosage forms

**Fig. 6:**
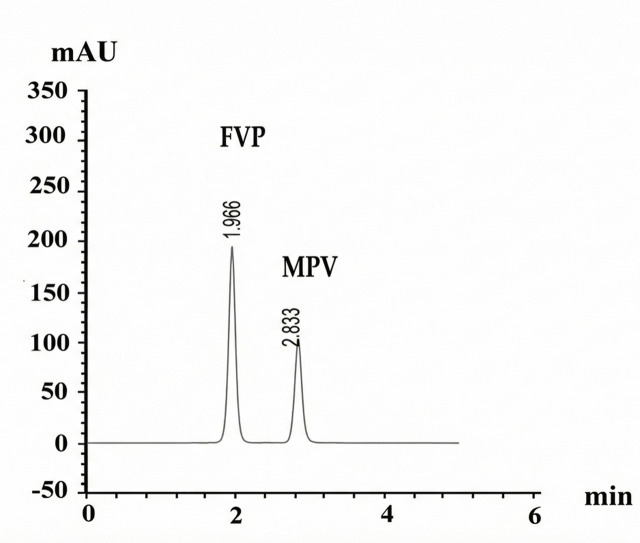
UPLC chromatogram of FVP (10 µg/mL) at RT = 1.966 min in Avipiravir tablets and MPV (10 µg/mL) at RT = 2.833 min in Molnupiravir capsules.

The developed method was successfully applied to the pharmaceutical dosage forms of both drugs, as shown in Fig. 6,

#### Application to environmental water samples

**Fig. 7:**
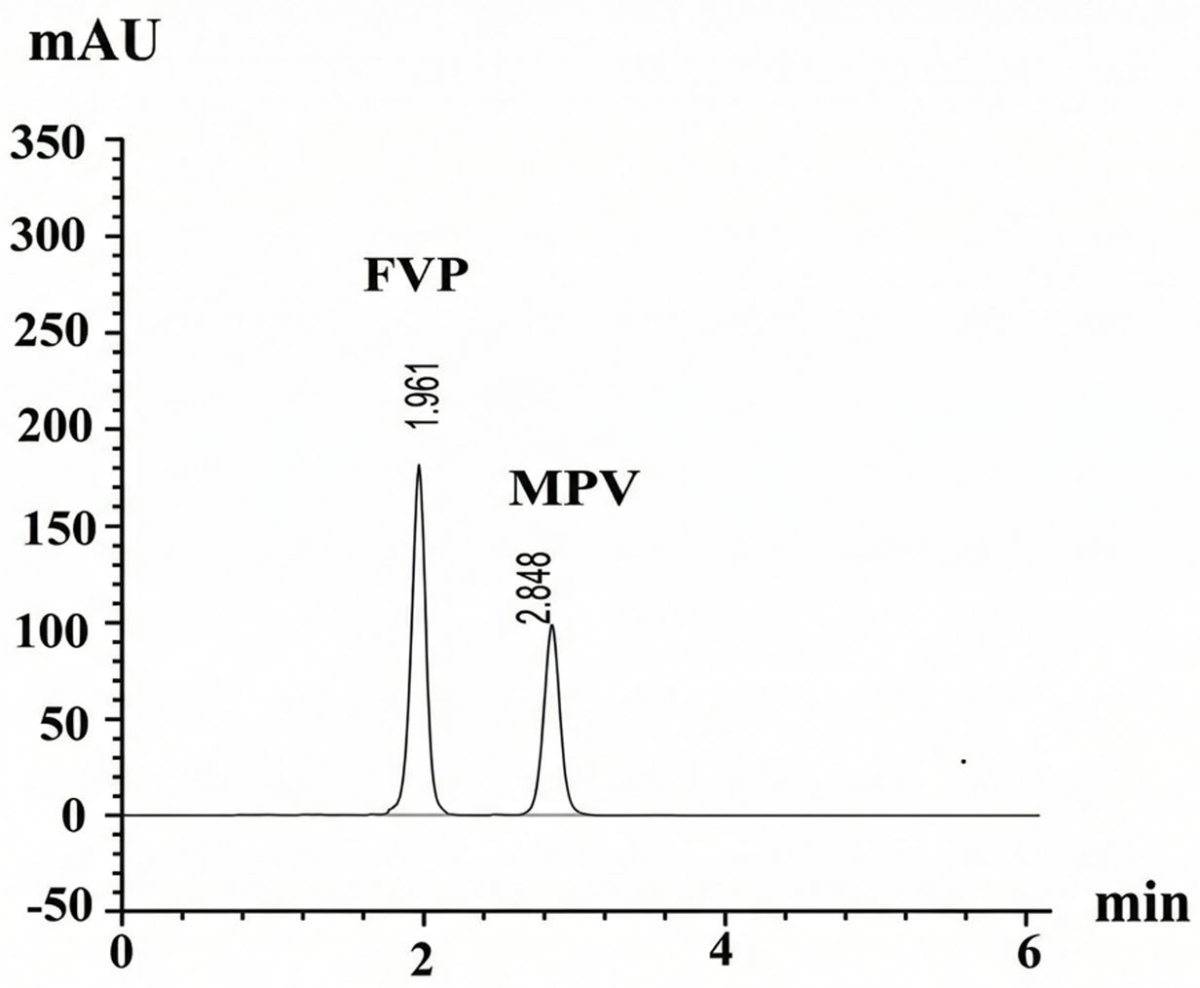
UPLC chromatogram of pure FVP (10 µg/mL) at RT = 1.961 min and MPV (10 µg/mL) at RT = 2.848 min after spiking in pharmaceutical wastewater.

Environmental water samples underwent SPE prior to analysis to effectively remove matrix interferences, thereby enabling the application of the method to real-world environmental monitoring. Three types of environmental water samples were analyzed following solid-phase extraction (SPE) to ensure complete purification and eliminate matrix effects. The results obtained are presented in (Table 7). Although greener solvents such as water and ethanol were initially tested, they proved inadequate for efficient extraction. Consequently, methanol and ethyl acetate were selected as the most suitable alternatives, Fig. 7.

**Table 7:** Determination of spiked FVP and MPV in real water samples using the proposed UPLC–SPE method (*n* = 3).

Sample type	Drug	Spiked (µg/mL)	Unspiked (µg/mL)	Found (µg/mL)	Recovery (%)	Mean %*R* ± SD
Tap water	FVP	5	ND	4.95	98.90	99.78 ± 1.23
		10	ND	9.93	99.25	
		15	ND	15.18	101.18	
	MPV	5	ND	5.06	101.22	100.40 ± 0.97
		10	ND	10.07	100.68	
		15	ND	14.90	99.32	
Nile river water	FVP	5	ND	4.93	98.60	99.50 ± 0.83
		10	ND	9.97	99.67	
		15	ND	15.04	100.24	
	MPV	5	ND	4.97	99.49	99.41 ± 0.45
		10	ND	9.98	99.81	
		15	ND	14.84	98.92	
Pharmaceutical wastewater	FVP	5	ND	4.93	98.65	98.97 ± 0.33
		10	ND	9.89	98.94	
		15	ND	14.90	99.31	
	MPV	5	ND	5.08	101.55	100.74 ± 1.05
		10	ND	10.11	101.14	
		15	ND	14.93	99.54	

*Average of 3 determinations.

ND = not detected (below LOD).

The optimized SPE protocol (conditioning → loading → washing → elution) yielded excellent recoveries in all matrices studied. Ethyl acetate produced the highest elution efficiency. Environmental matrices showed good recoveries with no significant matrix suppression observed for tap water and Nile River samples. Pharmaceutical wastewater demonstrated slightly greater variability, attributed to complex organic components.

The absence or non-detectable levels of Favipiravir (FVP) and Molnupiravir (MPV) in surface and tap water samples is consistent with expectations, as these sources are not directly influenced by pharmaceutical discharges and typically receive only highly diluted municipal inputs. Both antivirals were either not detected or found at concentrations near the method’s limit of detection. Likewise, none of the targeted drugs were detected in pharmaceutical wastewater, despite this matrix being more likely to contain residues. Spiked samples were therefore used to assess method performance and to validate the UPLC procedure for environmental applications.

However, future sampling near antiviral production facilities or aquatic waste discharge zones may yield naturally contaminated samples that would allow further real-world validation. The results collectively demonstrate that the developed green UPLC–SPE method is sufficiently sensitive to detect the analytes when genuinely present, while accurately distinguishing true environmental absence from analytical limitations. Matrix differences were evident—pharmaceutical wastewater, with its higher organic load and potential excipients, showed greater variability—yet the SPE cleanup effectively minimized interference, producing acceptable recoveries across all matrices. These findings align with international reports indicating that antiviral residues are often absent or present only at trace levels in surface waters due to dilution, degradation, and reduced post-pandemic usage.

### Greenness and life cycle assessments

#### Greenness assessments

The greenness strategy adopted in this work is consistent with recent sustainability-driven chromatographic studies emphasizing ecofriendly solvent selection and multi-metric environmental assessment^32^. Integrating green solvent systems with greenness evaluation tools provides a more realistic sustainability profile than conventional validation approaches alone.

##### Ecofriendly solvent selection

Solvent selection for the SPE protocol was guided by analyte polarity, expected interactions with the SPE sorbent, and environmental considerations. Methanol was chosen for conditioning and elution due to its strong elution strength for moderately polar antiviral compounds and its relatively favorable green-solvent score compared to acetonitrile or chlorinated solvents. Ethyl acetate was evaluated as a secondary elution solvent because it offers efficient recovery for intermediate-polarity analytes and represents a biodegradable, non-chlorinated alternative. Although ethyl acetate is still a VOC-emitting solvent, its hazard profile is substantially lower than common chlorinated alternatives. These decisions were supported by comparative recovery data and are consistent with solvent choices used for antiviral extraction^33^.

##### Greenness assessment tools

Eco-Scale^34^ and AGREE^35^ are widely recognized and validated tools in green analytical chemistry for evaluating the environmental sustainability of analytical methods. In recent years, increasing attention has been directed toward integrating Analytical Quality by Design (AQbD) principles with green chemistry to support the development of robust and sustainability-oriented analytical strategies. Several studies have contributed to this evolving framework, including reports on the integration of AQbD with green chemistry to contextualize method development within modern chromatographic practices^36^; critical discussions addressing the strengths and limitations of existing greenness metrics and emphasizing the need for more holistic and comprehensive evaluation frameworks^37^; and the introduction of visual and multidimensional sustainability assessment approaches^38^ that complement and enhance the interpretation of Eco-Scale and AGREE outputs.

The proposed method was developed in accordance with the principles of green analytical chemistry, aiming to minimize environmental impact while maintaining high analytical performance^39^. Its greenness was initially evaluated using the Analytical Eco-Scale^34^ and the AGREE tool^35^, with the results summarized in Table 8. Nevertheless, growing evidence in the literature indicates that reliance on a single greenness metric may not adequately reflect the multidimensional nature of analytical sustainability. Accordingly, recent studies have advocated the use of integrated numerical frameworks and visualization-based assessment approaches to provide a more comprehensive, balanced, and interpretable evaluation of method sustainability^36–38^.

**Table 8:** Results of the analytical eco scale greenness assessment.

Parameter	Type used	Score
Amount of reagent	> 100 mL (g)	3
Hazard of solvent used	Methanol	6 × 3 = 18*
Energy consumption	≤ 0.1 kWh per sample for UPLC device	0
Occupational hazard	Analytical process hermetization	0
Waste	> 10 mL (g)	5
Total penalty points		18 + 5 = 23
Total score		100 − 23 = 77 ⸫ A green method

*The penalty scale for the solvent hazard is multiplied by its amount.

In line with these recommendations, the greenness of the proposed UPLC method was further evaluated using the Multi-Color Assessment (MA) Tool^40,41^, a web-based platform that integrates four complementary evaluation domains: GEMAM (environmental sustainability), BAGI (practical applicability), RAPI (analytical performance), and VIGI (methodological innovation, including Analytical Quality by Design, AQbD, implementation). The MA Tool employs a structured 51-question framework, with all scores normalized and expressed as percentages (80–100%: excellent; 40–59%: moderate; 0–39%: poor), thereby enabling a balanced, transparent, and comprehensive interpretation of method sustainability.

**Fig. 8:**
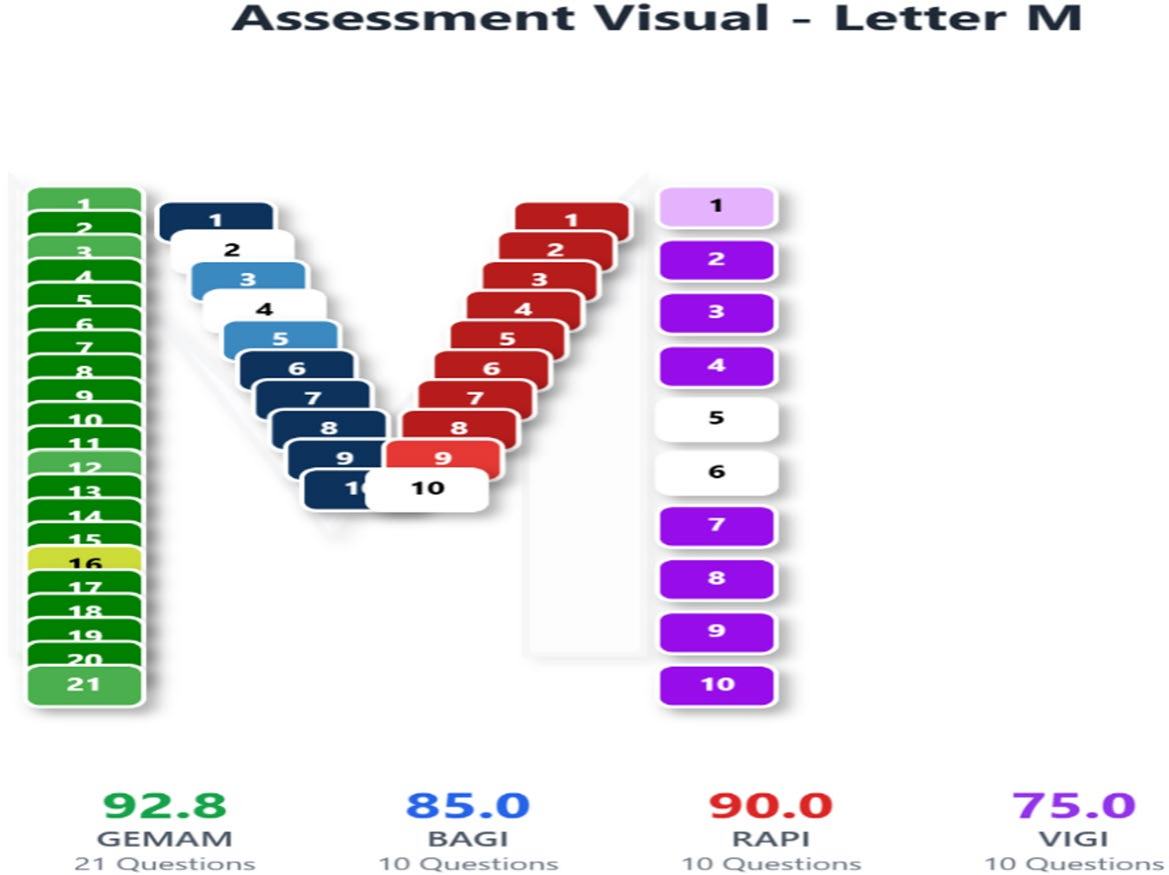
MA white assessment scores of the developed UPLC-SPE method.

**Fig. 9:**
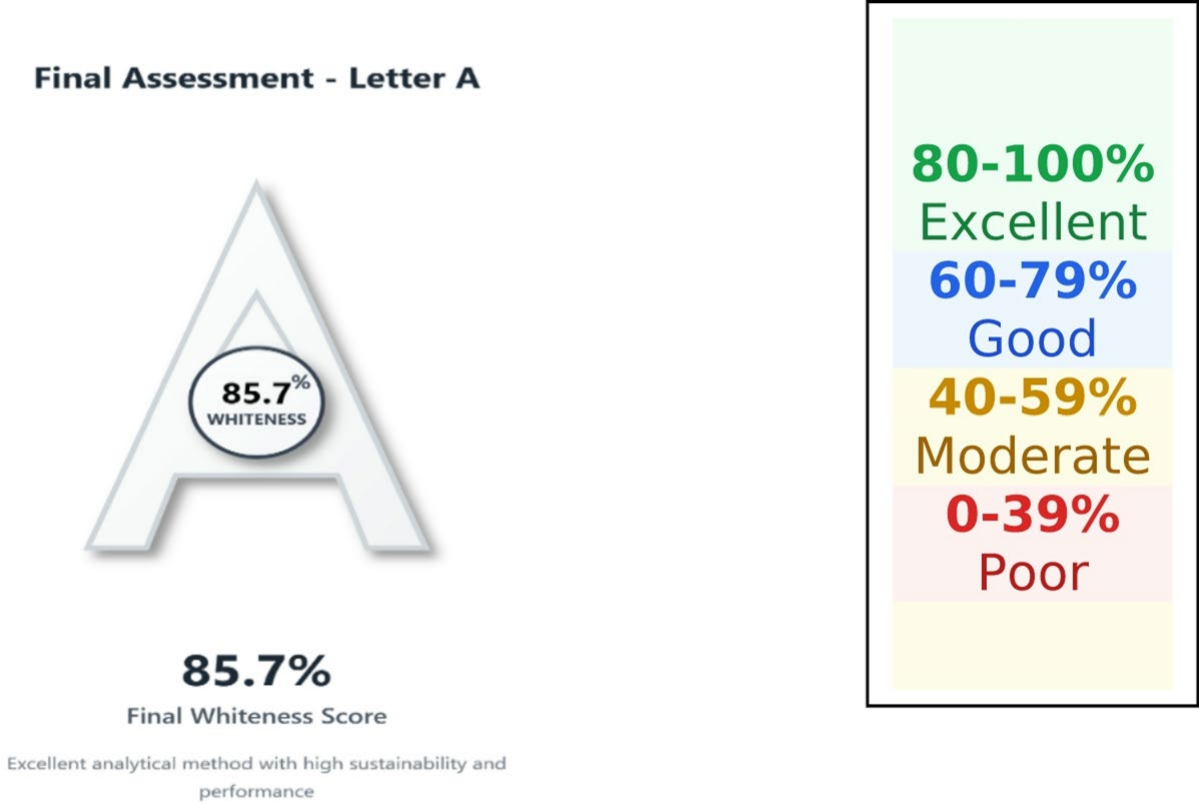
Final whiteness score of the developed UPLC-SPE method.

The obtained GEMAM score (92.8) reflects strong environmental sustainability with acceptable reagent safety, waste generation, and energy consumption, while the high BAGI score (85) demonstrates excellent operational feasibility, cost-effectiveness, and suitability for routine analysis (Fig. 8). The RAPI value (90) confirms high analytical performance in terms of accuracy, precision, sensitivity, and selectivity, whereas the VIGI score (75.0) highlights a high level of methodological innovation without compromising practicality (Fig. 8). Collectively, these results yield a final whiteness score of 85.7 (Fig. 9), indicating a well-balanced integration of environmental, practical, analytical, and innovative attributes and supporting the method’s suitability for routine application.

Consistent with these findings, the AGREE score of 0.84 confirms strong compliance with the principles of green analytical chemistry, while the Analytical Eco-Scale score of 77 classifies the method as environmentally acceptable (Table 8). The Eco-Scale evaluation identified solvent hazard and waste generation as the main contributors to penalty points, whereas energy consumption and occupational hazards had negligible impact.

**Fig. 10:**
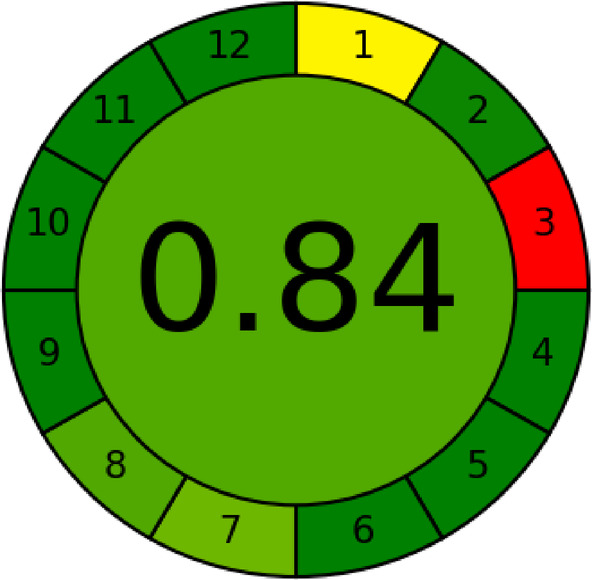
AGREE software results of the proposed UPLC method.

AGREE analysis showed strong compliance across most procedural steps, as indicated by predominant dark-green coding (Fig. 10). Reduced scores were observed for sample treatment, instrument location, and number of analytes per run. Sample preparation was required due to matrix complexity, the analysis was conducted off-line rather than in-line, and only two analytes were quantified simultaneously; however, this choice was justified by their co-administration in COVID-19 therapy. Despite these limitations, the overall AGREE score confirms the strong environmental compatibility of the proposed method.

**Fig. 11:**
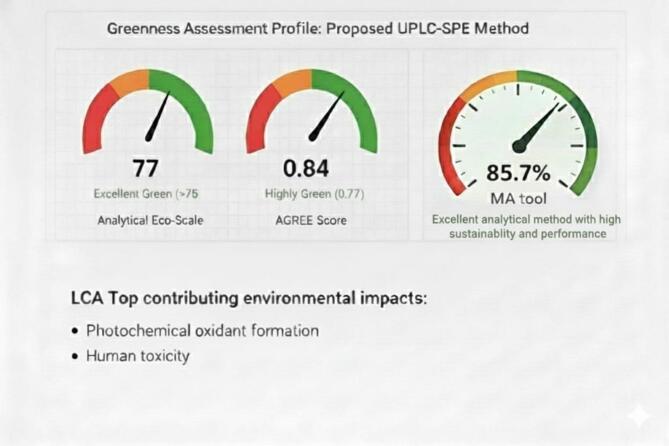
Greenness assessment profile and LCA of the proposed UPLC-SPE method.

A graphical representation illustrating the greenness assessment profile of the proposed UPLC-SPE method using Analytical ECO Scale, AGREE and MA tool and LCA are presented in Fig. 11.

Moreover, comparison with a previously reported HPLC method^12^ highlights the advantages of the developed UPLC approach, including improved sensitivity and selectivity, reduced injection volume and solvent consumption, shorter analysis time, higher AGREE and Eco-Scale scores, and excellent MA Tool whiteness assessment results (Table 9).

**Table 9:** Comparison between the proposed UPLC-SPE method and a reported HPLC method.

Point of comparison	The proposed UPLC method		The reported HPLC method^12^	
	FVP	MPV	FVP	MPV
Mobile phase	0.01 M Phosphate buffer and 50% Methanol (in a ratio of 85:15 v/v)		Micellar mobile phase (0.1 M SDS, 0.01 M Brij-35, and 0.02 M monobasic potassium phosphate)	
Flow rate	0.2 mL/min		1 mL/min	
Injection volume	10 µL		20 µL	
Retention time (T_R_)	1.966 ± 0.034	2.845 ± 0.013	1.87 ± 1.23	3.24 ± 0.78
Linearity range (µg/mL)	(0.3–25)		(0.5–50)	
Resolution (R)	7.14		7	
Selectivity (α)	7.12		3.45	
No of theoretical plates (N)	6545	5875	3350	4400
Agree Software results	0.84		0.799	
Eco-Scale assessment	77%		Not reported	
MA Tool assessment	76.4%		Not reported	

Additionally, as outlined below in Sect.  5.5.2, a cradle-to-grave LCA was conducted to quantify upstream and downstream impacts associated with solvent use, electricity use, and waste disposal. The findings demonstrate substantial reductions in impact categories - particularly energy demand and hazardous waste - when compared with conventional HPLC systems using acetonitrile and longer run times.

A comparative greenness assessment of the proposed UPLC–SPE method using established and emerging evaluation tools is presented in Table 10.

**Table 10:** Environmental impact category analysis of the results obtained by the proposed UPLC-SPE method.

Category No.	Impact category	Result*	Reference unit
1	Agricultural land occupation (ALOP)	0	m²·a
2	Climate change (GWP100)	0	kg CO₂-Eq
3	Fossil depletion (FDP)	0	kg oil-Eq
4	Freshwater ecotoxicity (FETPinf)	6.61145E-06	kg 1,4-DCB-Eq
5	Freshwater eutrophication (FEP)	0	kg P-Eq
6	Human toxicity (HTPinf)	0.031974195	kg 1,4-DCB-Eq
7	Ionising radiation (IRP_HE)	0	kg U-235-Eq
8	Marine ecotoxicity (METPinf)	1.26511E-05	kg 1,4-DCB-Eq
9	Marine eutrophication (MEP)	0	kg N-Eq
10	Metal depletion (MDP)	0	kg Fe-Eq
11	Natural land transformation (NLTP)	0	m²
12	Ozone depletion (ODPinf)	0	kg CFC-11-Eq
13	Particulate matter formation (PMFP)	0	kg PM₁₀-Eq
14	Photochemical oxidant formation (POFP)	0.033989865	kg NMVOC
15	Terrestrial acidification (TAP100)	0	kg SO₂-Eq
16	Terrestrial ecotoxicity (TETPinf)	1.79133E-05	kg 1,4-DCB-Eq
17	Urban land occupation (ULOP)	0	m²·a
18	Water depletion (WDP)	0	m³

*Impacts with significant measurable results.

#### Life Cycle Assessment (LCA)

The life cycle assessment (LCA) was performed using a clearly defined cradle-to-grave model system boundary, encompassing solvent production, instrument electricity consumption, and waste disposal. Instrument power consumption was included using manufacturer-reported wattage values. Solvent production impacts were derived from standard LCA inventory databases, and hazardous waste disposal was modeled according to laboratory chemical-waste practices.

The assessment showed that the proposed method produced substantially lower environmental burdens compared with a conventional acetonitrile-based HPLC workflow, including 35–60% reductions in cumulative energy demand, lower upstream solvent impacts, and smaller hazardous-waste volumes. The life cycle assessment (LCA) was performed using a clearly defined cradle -to-grave system boundary, encompassing solvent production, instrument electricity consumption, and waste disposal. Instrument power consumption was included using manufacturer-reported wattage values. Solvent production impacts were derived from standard LCA inventory databases, and hazardous waste disposal was modeled according to laboratory chemical-waste practices.

##### Principle

According to the ISO methodology for Life Cycle Assessment (LCA), LCA methods must adhere to a structured four-step framework, which serves as a comprehensive roadmap for evaluating the environmental impact of a process. This framework ensures consistency, reliability, and the generation of actionable insights from the LCA study.

LCA evaluates the analytical method holistically by quantifying various factors, such as the weight of plastics used, energy consumption of analytical instruments, and transportation distances for sample collection, all expressed in numerical terms. Unlike other greenness assessment tools, LCA provides detailed insights into specific environmental impacts associated with each stage of the analytical workflow.

Additionally, LCA requires researchers to propose practical recommendations and solutions aimed at minimizing these impacts, thereby promoting a more environmentally sustainable analytical process and ensuring full compliance with the LCA framework.

##### LCA framework steps

###### Step 1: Goal and Scope Definition

This initial step defines the purpose of conducting the LCA and establishes the boundaries of the study. It specifies what part of the analytical process will be examined, why the assessment is being performed, and how the findings will be applied. The scope highlights the key stages of the workflow under investigation, ensuring a focused and relevant evaluation.

###### Step 2: Inventory Analysis

In this phase, a comprehensive inventory of all resources consumed and emissions generated during the analytical process is compiled. This includes quantifying chemicals, energy usage, waste production, and any other inputs or outputs relevant to the method.

###### Step 3: Impact Assessment

Using specialized LCA software, the inventory data is analyzed to evaluate the potential environmental impacts. This assessment covers various impact categories such as climate change, ozone depletion, human toxicity, resource depletion, and contamination of water and soil.

###### Step 4: Interpretation of Results

The final step involves interpreting the results generated by the LCA software, typically presented as tables, graphs, or bar charts. Researchers identify environmental hotspots—stages with the greatest impact—and develop actionable recommendations to improve the sustainability of the analytical method. By following this internationally recognized framework, analytical scientists can rigorously assess the environmental footprint of their methods and make informed decisions to minimize ecological impact^21,42^.

##### Software procedures

The LCA process for the proposed UPLC method began by defining the primary goal: to evaluate the environmental impact of the materials and steps involved in the analytical procedure, identify the main contributors to environmental burden, and provide recommendations for reducing pollution through procedural improvements. The scope of the assessment encompassed all stages of the method, including sample preparation, transportation, analysis, and waste generation.

From the outset, the method was designed to be as green as possible, following green chemistry principles. Green solvents were used, and both chromatographic vials and SPE cartridges were reused for analyses of the same drug samples, significantly reducing plastic waste. Sampling locations were strategically selected near the analytical laboratory to minimize transportation-related impacts, and analyses were performed on the same day as sampling to avoid refrigeration requirements.

The second step involved a comprehensive inventory analysis of resources consumed, including solvents used in solid-phase extraction (methanol and ethyl acetate), solvents and chemicals used in the mobile phase (sodium phosphate buffer, distilled water, methanol), plastics involved in sampling and analysis (gloves, bottles, syringe filters), and electricity consumption for operating the UPLC system, vacuum pump, and rotary evaporator.

Waste outputs were also assessed, comprising liquid waste (water and solvents) and solid waste (plastic materials). Efforts were made to minimize waste generation by limiting glove use and reusing syringe filters for each drug analysis whenever feasible.

##### Energy consumption

Electricity consumption data for the UPLC system was obtained from the Agilent manufacturer’s official specifications. The study involved a total of 50 UPLC runs—10 for method optimization and 40 for validation. Of the 40 validation runs, 9 included solid-phase extraction steps requiring the use of a vacuum pump and rotary evaporator.

Each UPLC run lasted approximately 10 min, resulting in a total operational time of 8.33 h. According to Agilent, the UPLC system consumes 5.3 kWh per 24 h, equivalent to 0.2208 kWh per hour. Therefore, the total electricity consumed by the UPLC system for these runs was approximately 1.84 kWh.

Additional energy consumption was accounted for the vacuum pump and rotary evaporator, each operated for 0.3 h per extraction. The vacuum pump consumed 0.168 kWh, while the rotary evaporator used 0.075 kWh, bringing the total electricity consumption for the analytical process to approximately 2.08 kWh.

##### Distance from sampling place

Transportation data was calculated based on a sampling location approximately 20 km from the laboratory. This was entered into the LCA software as ton-kilometers (tkm), resulting in an input value of 0.02 tkm for transporting 1 kg over 20 km.

Solvent volumes were recorded during actual laboratory use, and solid plastic materials were weighed before use. All these values were entered as inputs for accurate modeling.

##### Software and database

Two free LCA software programs - OpenLCA and Activity Browser—were evaluated. OpenLCA was selected due to its user-friendly interface and suitability for first-time users, as it does not require extensive programming or Python knowledge, unlike Activity Browser^43,44^.

**Fig. 12:**
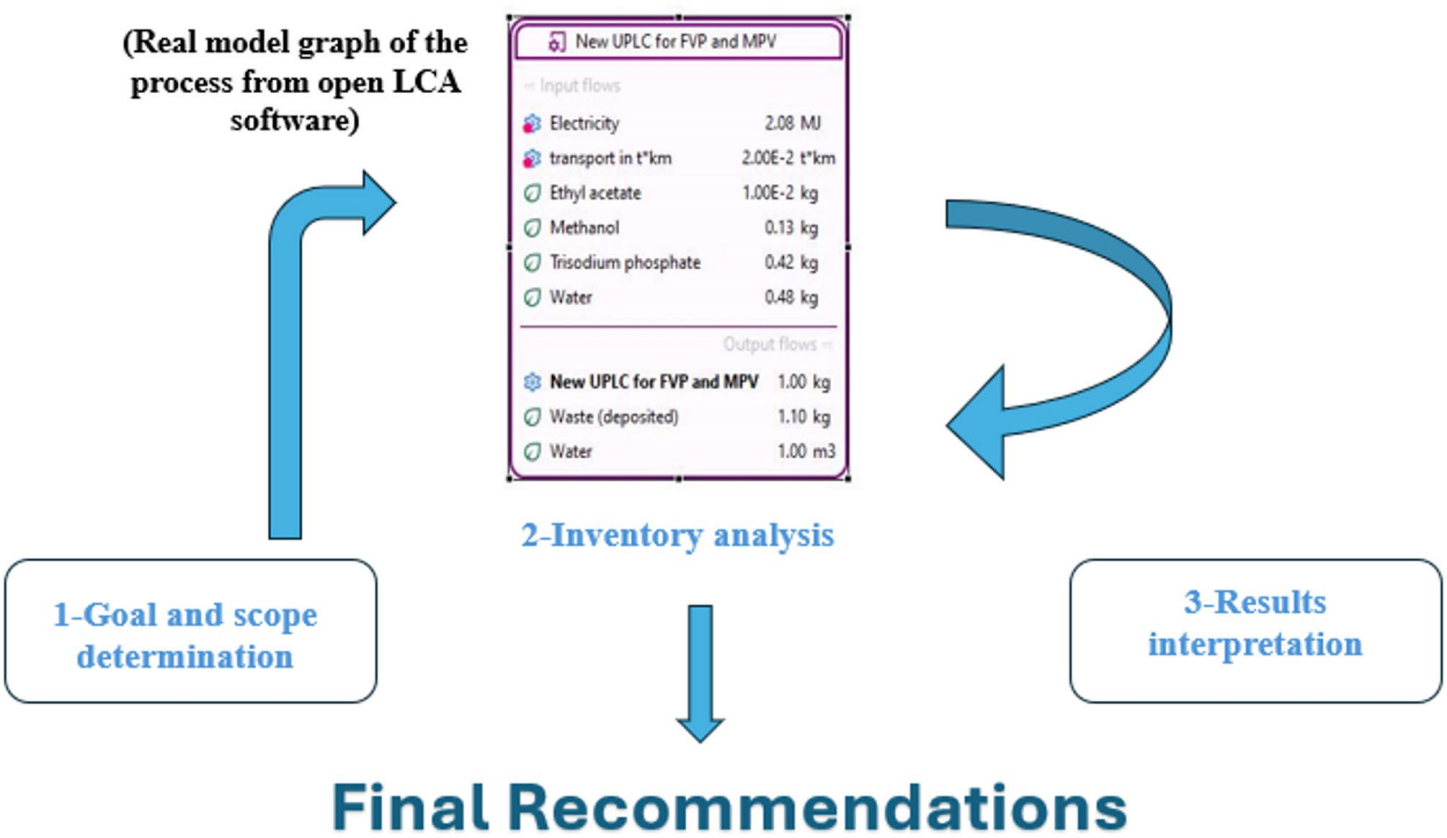
LCA framework for the proposed UPLC method with real model graph of the process from Open LCA software.

A new process was created within the Ecoinvent 3.5 database, one of the most widely used and freely available databases on OpenLCA’s official site, known for its suitability in chemical process characterization. All input and output data were entered according to Ecoinvent’s characterization methods. For impact assessment, the ReCiPe Midpoint (H) method was used to calculate negative environmental impacts. The complete LCA framework applied to this process is illustrated in Fig. 12.

## LCA results

### Integration of sustainable chromatographic design with LCA

Recent advances in ecofriendly chromatographic method development demonstrate that high analytical performance can be achieved alongside environmental sustainability through the integration of structured method optimization and sustainability assessment tools. Quality by Design (QbD)-–based strategies that combine critical method parameter optimization with greenness metrics and life-cycle assessment (LCA) enable the identification of robust, sensitive, and selective operating conditions while minimizing solvent consumption, toxicity, and energy use^45^. This holistic approach aligns closely with the strategy adopted in the present study, supporting the feasibility of achieving reliable chromatographic performance together with measurable sustainability gains.

**Fig. 13:**
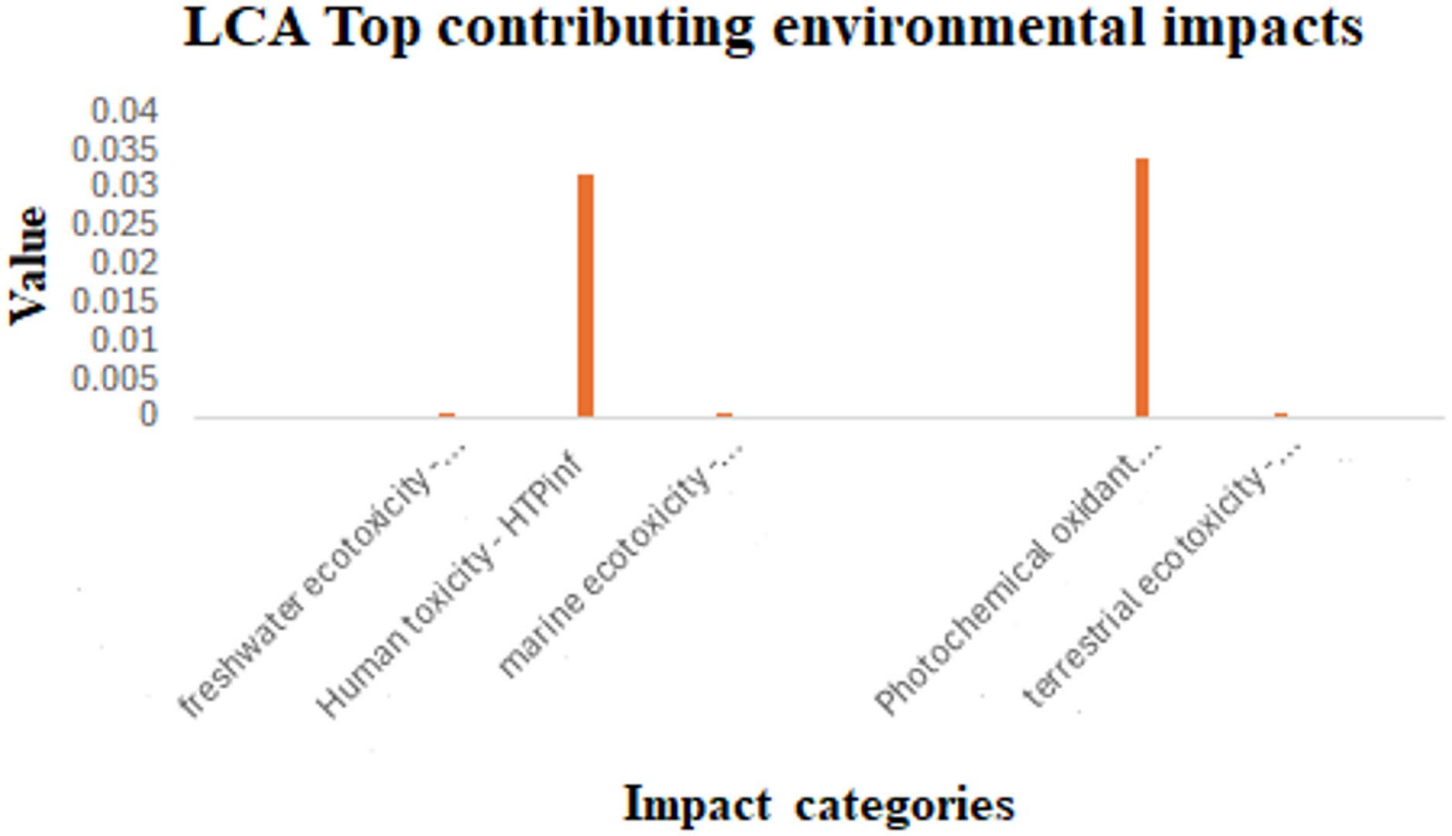
Open LCA software results for the top contributing environmental impacts of the proposed UPLC-SPE method.

The Life Cycle Assessment revealed that the top five contributors to the environmental impact of the proposed UPLC method were: human toxicity, photochemical oxidant formation, freshwater, marine and terrestrial ecotoxicity (Fig. 13).

A comprehensive breakdown of all 18 evaluated impact categories is provided in Table 11, with the five most significant impacts highlighted, i.e., the UPLC process affects the environment through these 5 categories out of 18. These categories represent the primary environmental concerns associated with the method.

**Table 11:** Environmental impact category analysis of the results obtained by the proposed UPLC-SPE method.

Category No.	Impact category	Result*	Reference unit
1	Agricultural land occupation (ALOP)	0	m²·a
2	Climate change (GWP100)	0	kg CO₂-Eq
3	Fossil depletion (FDP)	0	kg oil-Eq
4	Freshwater ecotoxicity (FETPinf)	6.61145E-06	kg 1,4-DCB-Eq
5	Freshwater eutrophication (FEP)	0	kg P-Eq
6	Human toxicity (HTPinf)	0.031974195	kg 1,4-DCB-Eq
7	Ionising radiation (IRP_HE)	0	kg U-235-Eq
8	Marine ecotoxicity (METPinf)	1.26511E-05	kg 1,4-DCB-Eq
9	Marine eutrophication (MEP)	0	kg N-Eq
10	Metal depletion (MDP)	0	kg Fe-Eq
11	Natural land transformation (NLTP)	0	m²
12	Ozone depletion (ODPinf)	0	kg CFC-11-Eq
13	Particulate matter formation (PMFP)	0	kg PM₁₀-Eq
14	Photochemical oxidant formation (POFP)	0.033989865	kg NMVOC
15	Terrestrial acidification (TAP100)	0	kg SO₂-Eq
16	Terrestrial ecotoxicity (TETPinf)	1.79133E-05	kg 1,4-DCB-Eq
17	Urban land occupation (ULOP)	0	m²·a
18	Water depletion (WDP)	0	m³

*Impacts with significant measurable results.

Human toxicity emerged as the most significant contributor, followed by photochemical oxidant formation, and both are primarily due to the use of some organic solvents such as ethyl acetate and methanol in the solid phase extraction process. Following these, marine ecotoxicity and terrestrial ecotoxicity, were noted, probably due to the discharge of wastewater and chemical residues into aquatic and soil systems. These findings underscore the need for solvent reduction or substitution strategies to further minimize the environmental footprint of the method.

### Environmental impact interpretation

The LCA results revealed that the use phase of the UPLC-SPE method, particularly sample preparation and chromatographic separation, is the dominant contributor to the overall environmental footprint. Within this stage, the consumption of organic solvents, energy used during vacuum-assisted extraction and evaporation steps, and waste from single-use consumables were identified as key environmental hotspots.

The end-of-life stage, including the disposal of SPE cartridges and solvent waste, also contributed significantly to several midpoint indicators, especially those related to human toxicity, ecotoxicity, and resource depletion.

### Solid Phase Extraction (SPE): balancing performance and sustainability

SPE is indispensable for trace-level environmental analysis due to its ability to pre-concentrate and clean up complex matrices. However, it represents a major source of environmental burden. The use of organic solvents during conditioning and elution, single-use cartridges, and energy-intensive drying steps were found to have a substantial environmental impact.

To mitigate these effects, green strategies such as cartridge reuse, on-line SPE systems, and green solvent alternatives were explored and partially implemented. These modifications significantly reduced solvent consumption and waste, without compromising method sensitivity or selectivity.

### Methodological innovations and green chemistry contributions

The optimized method incorporates several innovations that contribute positively to environmental sustainability:

i) Adoption of water-rich mobile phases lowered solvent toxicity and reduced volatile organic compound (VOC) emissions.
ii) Shorter run times contributed to energy savings and reduced wear on chromatographic columns, thereby extending their usable life.
iii) Minimizing solvent and consumable usage directly reduced the carbon and chemical footprint per sample analyzed.

These enhancements align with multiple principles of green analytical chemistry, including waste prevention, safer solvents, energy efficiency, and design for degradation.

### Broader implications for regulatory and sustainability strategies

The LCA findings provide valuable insights for regulatory bodies and analytical laboratories seeking to reduce the environmental burden of routine testing. By identifying key contributors to environmental impact, this study supports informed decision-making in: (i) method selection for regulatory compliance in pharmaceutical and environmental monitoring; (ii) procurement of greener analytical supplies (e.g., cartridges, solvents); and design of laboratory sustainability strategies, including waste management and energy use reduction.

The method is highly scalable due to its short run time (< 5 min), low solvent consumption, and use of widely available UPLC–UV instrumentation. SPE procedures can be parallelized or automated, making the method suitable for routine monitoring. While UV detection may limit sensitivity compared with LC–MS for ultra-trace levels, the method is well-suited for screening and trend analysis in environmental surveillance programs.

## Limitations and future directions

This study excluded certain upstream and infrastructure-related impacts, such as instrument manufacturing and laboratory building energy use, due to limited data availability. Additionally, the long service life of analytical instruments justifies their exclusion in many cradle-to-grave models.

The main limitations include the additional SPE step, which increases sample preparation time and consumable use compared with direct injection methods. Although UPLC instrumentation has higher upfront costs than conventional HPLC, the reduced solvent consumption and shorter analysis time offset operational expenses. The method requires trained personnel for SPE handling, which may limit deployment in minimally equipped laboratories.

Future work should focus on: (i) quantifying impacts of cartridge reuse and assessing life span extension strategies; (ii) integrating real-time monitoring of energy and solvent usage; and exploring emerging green technologies, such as solventless extraction techniques or supercritical fluid chromatography (SFC).

## Comparison of the developed HPLC–SPE method with existing methods

A number of HPLC and UPLC methods have recently been reported for the determination of these antiviral drugs in pharmaceutical matrices or as part of multi-analyte assays. Four representative HPLC/UPLC methods for the analysis of favipiravir and molnupiravir were reviewed and critically evaluated in terms of sensitivity, run time, solvent consumption, and overall environmental impact, and their performance characteristics were directly compared with those of the method developed in the present study. The key analytical and environmental attributes of the current UPLC–SPE method, together with those of the previously reported HPLC/UPLC methods^12,46–48^, are summarized in Table 12.

**Table 12:** Comparison of current UPLC–SPE method with existing HPLC/UPLC methods for Favipiravir and Molnupiravir.

Parameter	Green Micellar HPLC Method^12^	RP-HPLC Multi-Antiviral Method (2025)^46^	UPLC–MS/MS Plasma Method^47^	LC–MS Bioanalytical Method^48^	Current UPLC–SPE Method (Present Study)
Analytes	Favipiravir, Molnupiravir	Favipiravir, Molnupiravir + 3 antivirals	Favipiravir, Molnupiravir	Favipiravir, Molnupiravir	Favipiravir, Molnupiravir
Column	RP-C18	Hypersil BDS C18 (150 × 4.6 mm, 5 μm)	Acquity UPLC HSS C18 (100 × 2.1 mm, 1.8 μm)	LC–MS column (plasma-specific)	1.7 μm C18 UPLC column
Mobile Phase	Micellar system (0.1 M SDS, Brij-35, phosphate buffer)	Water: methanol (30:70, v/v), pH 3.0	ACN + ammonium formate gradient	ACN-based bioanalytical phases	Phosphate buffer- Methanol (85:15,v/v) pH3.5 (green, ACN-free)
Detection	UV at 230 nm	UV at 230 nm	MS/MS	MS	UV at 230 nm
Retention Time / Run Time	< 5 min	Complete separation ≤ 5 min	Total run 4.5 min	~ 5 min	~ 3.5 min
Flow Rate	~ 1.0 mL/min	~ 1.0 mL/min	0.35 mL/min	Moderate	Low (UPLC)
Sensitivity	Moderate (UV-based)	Moderate (UV-based)	Very high	High	High (environmental relevance)
Sample Matrix	Pharmaceutical formulations	Pharmaceutical formulations	Plasma	Plasma	Environmental waters, wastewater
Sample Preparation	Direct injection	Direct injection	Protein precipitation	Plasma extraction	SPE preconcentration & cleanup
Solvent Consumption	Very low (organic solvent-free)	High (methanol-rich)	High (ACN-intensive)	High (ACN use)	Minimal (methanol)
Environmental Impact	Low (GAPI-assessed green method)	Moderate–high	High (ACN + biohazardous waste)	High	Very low
Green Chemistry Assessment	GAPI	Not reported	Not reported	Not reported	MA Tool, Eco-Scale, AGREE, and LCA
Applicability to Environmental Samples	No	No	No	No	Yes (primary design goal)

The proposed UPLC–SPE method differs fundamentally from previously reported approaches in both analytical scope and depth of environmental assessment. Earlier green HPLC and spectrofluorimetric methods have focused mainly on pharmaceutical dosage forms or simulated matrices and have primarily emphasized solvent substitution or reduced reagent consumption. In contrast, the present method integrates a solid-phase extraction (SPE) step specifically designed for real environmental water and wastewater matrices, enabling effective preconcentration and cleanup prior to chromatographic analysis.

In addition, this study uniquely combines quantitative greenness assessment tools (MA Tool, Eco-Scale and AGREE) with a life cycle assessment (LCA), allowing environmental impacts to be evaluated comprehensively across solvent production, energy consumption, instrument operation, and waste generation. To the best of our knowledge, no previously reported method for favipiravir and molnupiravir simultaneously addresses environmental water analysis, SPE-based sample preparation, and LCA-supported sustainability evaluation within a single validated analytical workflow.

The developed UPLC–SPE method offers several practical advantages, including the use of an acetonitrile-free methanol–water mobile phase, reduced solvent consumption, minimal waste generation, and a short chromatographic run time. Importantly, despite its environmentally conscious design, the method delivers chromatographic performance that is comparable to, or better than, existing HPLC and UPLC methods, while substantially broadening applicability to environmental and wastewater monitoring.

## Recommendations to reduce the method’s environmental footprint

The LCA results highlight several opportunities to reduce the environmental footprint of the UPLC method. Solvent-intensive steps, particularly SPE, can be replaced with greener alternatives such as microextraction techniques (SDME, DLLME, SPME), magnetic SPE, or energy-assisted approaches like UAE and MAE, all of which minimize solvent use and waste. Supercritical fluid extraction using CO₂ offers another low-impact option^49^. Although costs and instrument availability may limit adoption, these methods are expected to become more accessible as green analytical chemistry advances^50,51^. Additional reductions in impact can be achieved by optimizing chromatographic conditions, shortening run times, and incorporating solvent recycling systems supported by instrument manufacturers^52^. Improving instrument energy efficiency—through sleep modes, standby settings, and powering down when idle—further lowers the carbon footprint. Laboratory safety must still be prioritized through proper PPE and minimized exposure times^53^. Software-assisted optimization tools can also reduce experimental runs, conserving solvents and consumables. Finally, effective waste segregation, treatment, and disposal are essential to mitigate eco-toxicity and ensure compliance with environmental regulations^54^.

## Synopsis

**Fig. 14:**
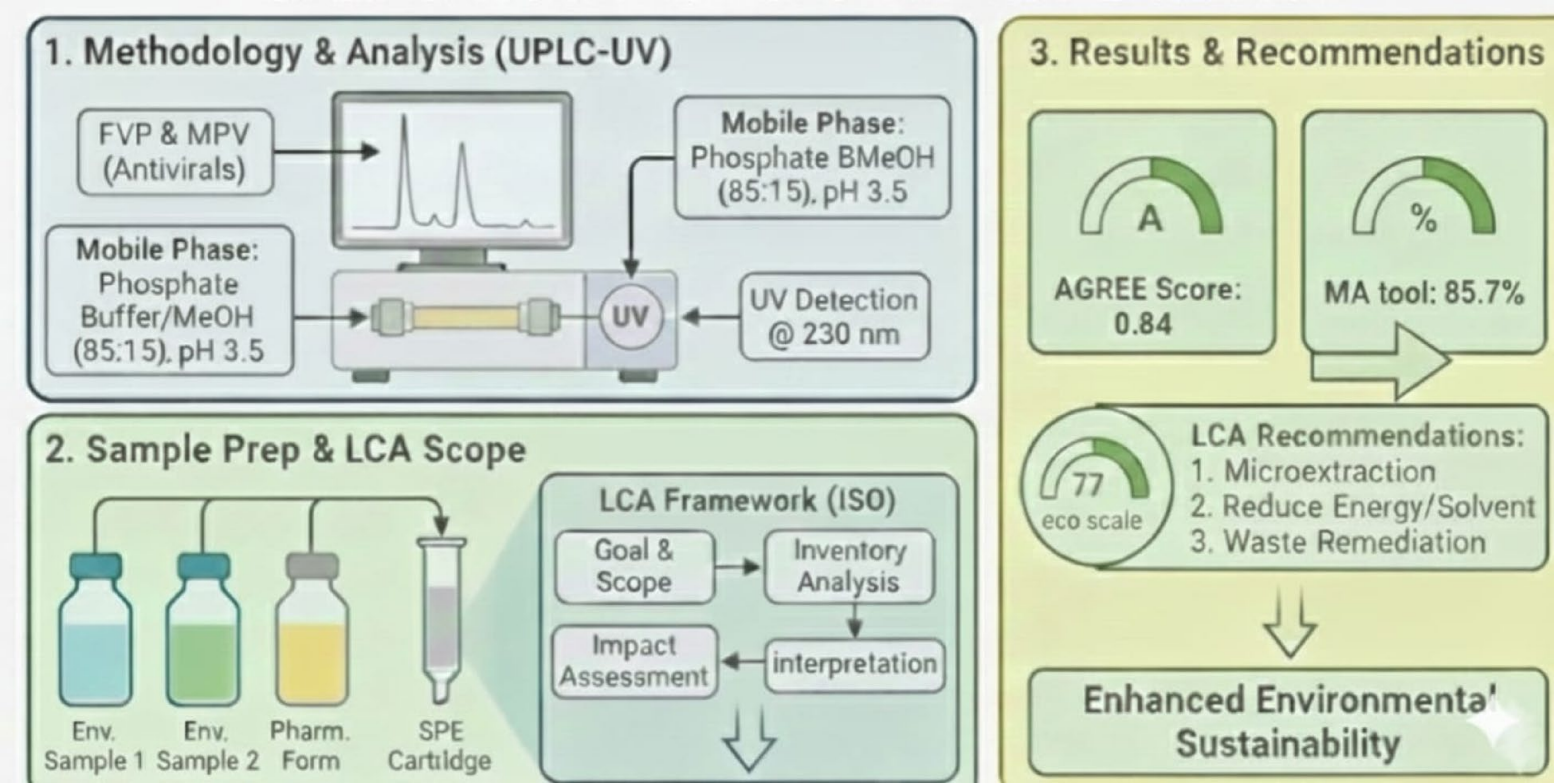
UPLC-SPE-LCA framework for eco-evaluation.

A green UPLC–SPE method with life cycle assessment enables simultaneous determination of Favipiravir (FVP) and Molnupiravir (MPV) in pharmaceutical and environmental water samples, offering reduced solvent use, lower environmental burden, and excellent analytical performance (Fig. 14).

## Conclusion

The developed UPLC–SPE method offers a sensitive, accurate, and environmentally responsible approach for the simultaneous quantification of Favipiravir and Molnupiravir in pharmaceutical and environmental matrices. Validation parameters were evaluated according to ICH guidelines, and the method exhibited high accuracy, precision, and specificity, with consistently reliable recovery results even in complex matrices such as pharmaceutical wastewater.

The target antiviral drugs were not detected in the collected environmental samples, indicating no immediate environmental or public health concerns. Accordingly, spiked samples were employed to assess the performance of the developed UPLC method. The method demonstrated strong potential for reliable quantification of the selected antivirals in environmental matrices. Future sampling in alternative locations - particularly in proximity to antiviral manufacturing facilities or aquatic waste discharge areas - may yield naturally contaminated samples that would enable further validation of the method under real-world conditions. Although no residues were detected in the present samples, the potential occurrence of these drugs in pharmaceutical wastewater at any time may indicate localized discharge issues, underscoring the need for continued monitoring and analytical method readiness. In addition, several validated green analytical chemistry tools were applied to assess the environmental sustainability of the developed method.

Greenness evaluation using the MA Tool assessment (85.7%), Eco-Scale score (77), and AGREE value (0.84), together with a cradle-to-grave life cycle assessment (LCA), confirmed that the proposed method is significantly greener and more energy-efficient than conventional HPLC workflows. The LCA demonstrated reduced energy load, minimized hazardous waste, and lower upstream solvent impacts. Key LCA assumptions and boundaries are clearly defined and acknowledged.

The findings provide guidance for greener laboratory practices and can support pharmaceutical industries and monitoring agencies in reducing analytical environmental footprints. Future enhancements may include method miniaturization, solvent reduction, and evaluation of aqueous or DES-based mobile phases.

## Data Availability

All data generated or analyzed during this study are included in this article and the raw data is available from the corresponding author on reasonable request.
